# Design and Implementation of Low-Voltage Tunable Capacitive Micro-Machined Transducers (CMUT) for Portable Applications

**DOI:** 10.3390/mi13101598

**Published:** 2022-09-26

**Authors:** Chirag Goel, Paul-Vahe Cicek, Alexandre Robichaud

**Affiliations:** 1 Department of Mechanical and Manufacturing Engineering, Manipal Institute of Technology, Manipal Academy of Higher Education, Manipal 576104, India; 2 Microtechnologies Integration & Convergence Research Group, Université du Québec à Montréal (UQAM), Montreal, QC H2X 3Y7, Canada; 3 Department of Applied Sciences, Université du Québec à Chicoutimi (UQAC), Chicoutimi, QC G7H 2B1, Canada

**Keywords:** capacitive micromachined ultrasonic transducers (CMUTs), medical imaging, MEMS, ultrasonic transducers

## Abstract

Capacitive micromachined ultrasonic transducers (CMUT) are MEMS-based transducers with advantages over conventional ultrasonic transducers, such as their small size, the ease of integration with semiconductor electronics, and batch fabrication. In this study, the effect of different membrane topologies on the displacement, resonant frequency, and output pressure of the CMUT membrane is investigated in the transmission mode in an air environment. A novel structural-support feature, the rocker stem, is introduced, where the membrane is weakly held to the substrate in order to minimize mechanical constraints. Four different CMUT topologies are designed and assessed to analyze the impacts of topological variations. A new CMUT array configuration is also designed to provide an approach for maximizing CMUT density. This study aims to contribute to efficient CMUT design and the determination of optimum structural parameters for portable applications in air.

## 1. Introduction

**Figure 1:**
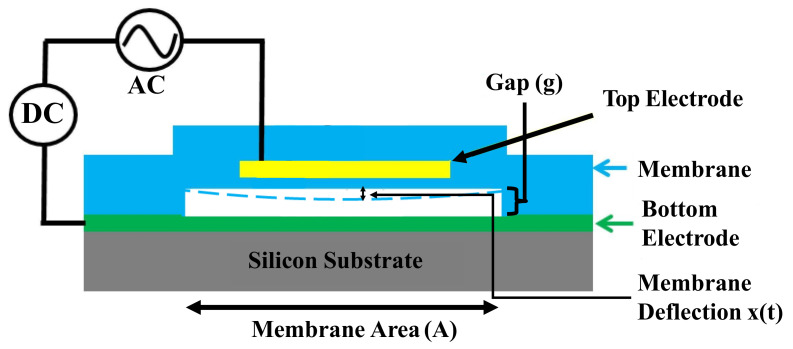
Basic CMUT schematic.

Capacitive micromachined ultrasonic transducers (CMUTs) are an attractive alternative to the traditional piezoelectric film-based transducers because of several advantages they may offer, such as larger bandwidth, increased sensitivity, efficient performance, IC-compatible fabrication and a variety of possible array configurations [1]. Relying on microelectromechanical systems (MEMS) technology, CMUTs have been studied in a large number of acoustic applications [2], which can be divided into two subsets: air-coupled applications, where the device is operated within air or general gaseous environments, and immersion applications where the device is located in, or in contact with, a liquid. CMUTs offer several advantages in air applications (e.g., ultrasonic ranging and non-destructive testing (NDT)) such as increased efficiency and accuracy, high dynamic range, improved tolerance to humidity or external particles, and resistance to chemicals [2]. For water applications such as underwater ranging, CMUTs provide a significant advantage due to their inherently wide bandwidth and smaller size for low-frequency operation. Medical imaging technology has also greatly benefited from the advances in CMUT [2]. There has indeed been a surge in interest for miniaturizing volumetric imaging devices for in-vivo applications such as intravascular imaging, surgical guidance and echography [3,4]. Wireless acoustic power transfer using capacitive ultrasonic transducers is gaining traction as a new technology to power IoT devices and for energy harvesting. Parametric-resonance-based capacitive transducers were designed in [5] which eliminated the need to apply DC bias voltage for its operation. Airborne ultrasound for wireless power transfer to mm-sized nodes was developed by [6], which delivers 50 μW at 0.88 m and receives 5 μW at a distance of 1.05 m. Micromachining processes relying on widespread techniques such as photolithography facilitate the affordable manufacturing of miniature CMUT arrays. CMUTs generate acoustic waves in a given medium through electrostatic force: a voltage signal comprising an AC component superimposed onto a DC bias is applied across the vibrating membrane and the fixed bottom electrode, resulting in a modulated electric force. This force drives the vibration of the membrane, in turn producing an acoustic wave at the same frequency as that of the AC signal [2]. A regular CMUT cell consists of a membrane (top electrode), an air or vacuum gap, and a bottom electrode, as shown in Figure 1 [7].

In order to integrate CMUTs in systems for portable ultrasonic applications, it is critical for the device to operate efficiently at a small bias voltage. Since the power transfer efficiency of a CMUT is maximal when the DC bias is as close as possible to the electrostatic collapse voltage, it would be desirable to work with a structure exhibiting a collapse voltage as low as possible above the desired bias [8]. The required bias voltage can be controlled by tuning various design parameters such as electrode area, membrane stiffness and the initial gap size between the electrodes. However, in general, bias-voltage optimization possibilities are limited when working with a clamped deformable body structure such as those typically achievable through MEMS technologies. Although collapse voltage and resonant frequency could easily be decreased by increasing the CMUT membrane (reducing effective stiffness, increasing electrostatic force), this would result in a larger CMUT element size, limiting how compact an array could be made. Therefore, it is worthwhile to devise a new approach to achieve a compact CMUT element with a low resonant frequency and a low collapse voltage. The work in [9] presented a reduced CMUT structure with a relatively low bias voltage and a high frequency for air-coupled as well as immersed applications; it demonstrated a four-fold reduction in the bias voltage for a constant membrane displacement. Recently, collapse-mode operation CMUTs have gained a lot of attention owing to their increased electric field strength which results in very high pressure output and better efficiency [10]. Frequency tunable CMUTs operating in collapse mode for ultrasound imaging applications were investigated in [11], where the operating frequency was tuned from 8.7 MHz to 15.3 MHz by varying the bias voltage from 50 V to 170 V, with a maximum sensitivity of 52 kPa/V. However, collapse-mode CMUTs have been shown to be prone to dielectric charging and subsequent reliability issues [1]. In a study [12], it was found that resonant frequency decreased by 20% when constraints on the membrane were decreased.

**Figure 2:**
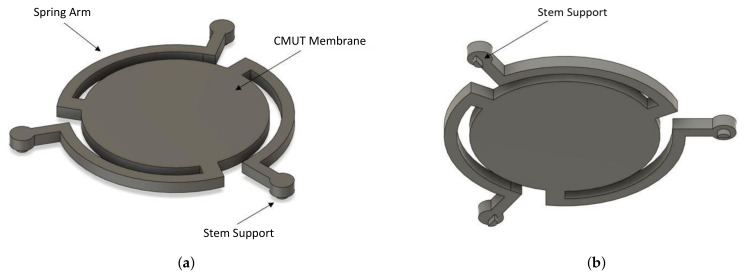
3D CAD of the designed CMUT A and B. (**a**) Top view; (**b**) bottom view.

In this work, a novel CMUT structure is introduced which exploits spring softening phenomena to increase frequency tunability at low bias voltages. This is made possible by the introduction of a spring arm structure holding the vibrating membrane through novel rocker support stems, as illustrated in Figure 2. A lower driving voltage is enabled by the reduced effective stiffness of the structure. Additionally, rocker stems minimize anchor losses through deformation and, thus, increase acoustic efficiency. The proposed approach further allows for smaller overall device dimensions for a given target operating frequency, which can help increase array density for beamforming applications. The proposed structures are designed to be readily implemented in a commercial surface micromachining technology, namely, the PolyMUMPs process, provided by MEMSCAP [13]. This paper studies and compares four alternate CMUT topologies in order to showcase the benefits of the novel structural features introduced. Applications targeted for this work include air-ultrasonic imaging (3D imaging and gesture recognition) [14], anemometry [15], thermoacoustic imaging, nondestructive testing, and ultrasonic flow metering [16].

The operation of the CMUT is explained in Section 2; methods of design and fabrication are presented in Section 3; finite element analysis (FEA) results and performance metrics are discussed in Section 4; Section 5 concludes.

## 2. Operating Principle

CMUT devices have two modes of operation: ultrasound generation and ultrasound detection. For ultrasound generation, the CMUT transmits an acoustic wave caused by the membrane vibrating in response to the alternating electrostatic force generated by AC potential applied across the top electrode (vibrating membrane) and the bottom electrode (fixed). The generated electrostatic force $F_{ele}$(*t*) is given by

(1) $$F_{ele}\left(t\right)=\frac{\varepsilon A}{2{\left(g-x\left(t\right)\right)}^{2}}{\left(V_{Signal}\right)}^{2}$$

(2) $$V_{Signal}=V_{DC}+V_{0}sin\left(\omega t\right)$$

where *A* is the membrane area, *ε* is the permittivity of free space, *g* is the initial gap size between the two electrodes, *x*(*t*) is the vertical displacement of the membrane due to the electrostatic force, and $V_{Signal}$ is the voltage signal which has both DC and AC components (Equation (2)). The AC component in the voltage signal is responsible for generating the acoustic wave.

When applying a voltage across the electrodes, the electrostatic force pulls the membrane toward the bottom electrode. This force is balanced by the mechanical restoring force exerted by the CMUT membrane structure, defined as

(3) $$F_{ele}\left(t\right)=K_{eff}x\left(t\right)$$

where $k_{eff}$ is the effective stiffness of the system, defined as the deflection that a structure exhibits under a given mechanical load. At any point in time, the deflection of the CMUT membrane, *x*(*t*), is determined by the equilibrium of the electrostatic and restorative forces. However, above a certain DC bias voltage, the electrostatic force overwhelms the mechanical restoring force, rendering the system unstable, and the membrane collapses onto the bottom electrode. This voltage is known as the pull-in or collapse voltage.

The collapse voltage can be calculated by solving the first derivative of $V_{DC}$ with respect to *x* at 0: the solution of the latter states that collapse always occurs when the displacement exceeds one third of the initial gap *g*:

(4) $$V_{DC}=\sqrt{\frac{2k_{eff}x}{\varepsilon A}}\left(g-x\left(t\right)\right)$$

(5) $$x=\frac{g}{3}$$

(6) $$V_{collapse}=\sqrt{\frac{8k_{eff}g^{3}}{27\varepsilon A}}$$

Equation (6) shows that, in order to decrease pull-in voltage, either the stiffness ($k_{eff}$) or the initial gap (*g*) of the system needs to be decreased. To lower the resonance frequency for a smaller CMUT structure, the stiffness of the system should be lowered, as per

(7) $$f_{res}=\frac{1}{2\pi }\sqrt{\frac{k_{eff}}{M_{total}}}$$

Since the initial gap size (*g*) is set by the thickness of the sacrificial layers in the PolyMUMPs process, it is important to minimize the stiffness of the vibrating CMUT membrane for improved sensitivity and lower resonance frequency for applications in portable devices. One of the most important parameters for a CMUT is its operating resonance frequency, as it determines the types of applications for which the device will be suitable. The acoustic energy loss of the ultrasonic wave is dependent on the type of medium, temperature, the distance of propagation and the frequency of the ultrasonic wave. For example, the amplitude of a sound wave is attenuated as some of the energy it carries is lost to friction and relaxation processes. Therefore, low-frequency sound waves are often preferred for longer ranges. However, a higher frequency ultrasonic wave may provide finer imaging resolution depth. As a result, an optimal resonant frequency range must often be selected [16]. The air gap between the vibrating membrane and the fixed electrode is another important parameter. The gap size should be small to optimize electromechanical-power transduction efficiency and reception sensitivity. However, a small gap also limits the maximum range of motion of the membrane before collapse, limiting the maximum amplitude of ultrasonic waves generated. As for the bandwidth performance of a CMUT, it is related to the mass of the membrane, as highlighted by the quality factor approximated by

(8) $$Q=\frac{m\omega_{0}}{R}$$

where *m* is the equivalent membrane mass, $\omega_{0}$ is the resonance frequency, and *R* is the radiation impedance.

In this study, a novel CMUT is developed with very low effective stiffness and anchor losses. Spring arms were designed to exert significantly less resistance (Equation (9)) compared to conventional straight, fixed supporting anchors (Equation (10)), through a combination of longer effective arm length because of curvature as well as the elimination of torsional and bending moments because of the rocker stems providing more degrees of freedom.

(9) $$k_{spring}=\frac{1}{\frac{\pi }{4}\frac{R^{3}}{EI}+\left(\frac{3\pi }{4}-2\right)\frac{R^{3}}{GJ}}$$

(10) $$k_{beam}=\frac{3EI}{L^{3}}$$

where *I* is the moment of inertia, *G* is the modulus of rigidity, *E* is the Young’s modulus, *J* is the torsional rigidity, *δ* is the deflection of the spring elements, *L* is the length of the beam, *F* is the force on the element, and *R* is the radius of curvature. Furthermore, CMUT with rocker stems reduce anchor mechanical coupling between the CMUT structure and the surface on which the anchors rest. The greater membrane displacement resulting from the lower mechanical losses increases the output pressure for the same DC bias and AC signal voltages [17,18].

The deflection of the quarter-circular beam at the free end is given by [19]

(11) $$\delta =\frac{\pi }{4}\frac{FR^{3}}{EI}+\left(\frac{3\pi }{4}-2\right)\frac{FR^{3}}{GJ}$$

Four alternate CMUT topologies were designed in this study to assess and compare the impact of the spring-arm geometry and anchoring method, as detailed in Table 1. The simulation results of the four topologies provide the performance characteristics useful to assess the incidence of the novel features introduced.

## 3. Methods

### 3.1. PolyMUMPS Fabrication

**Figure 3:**
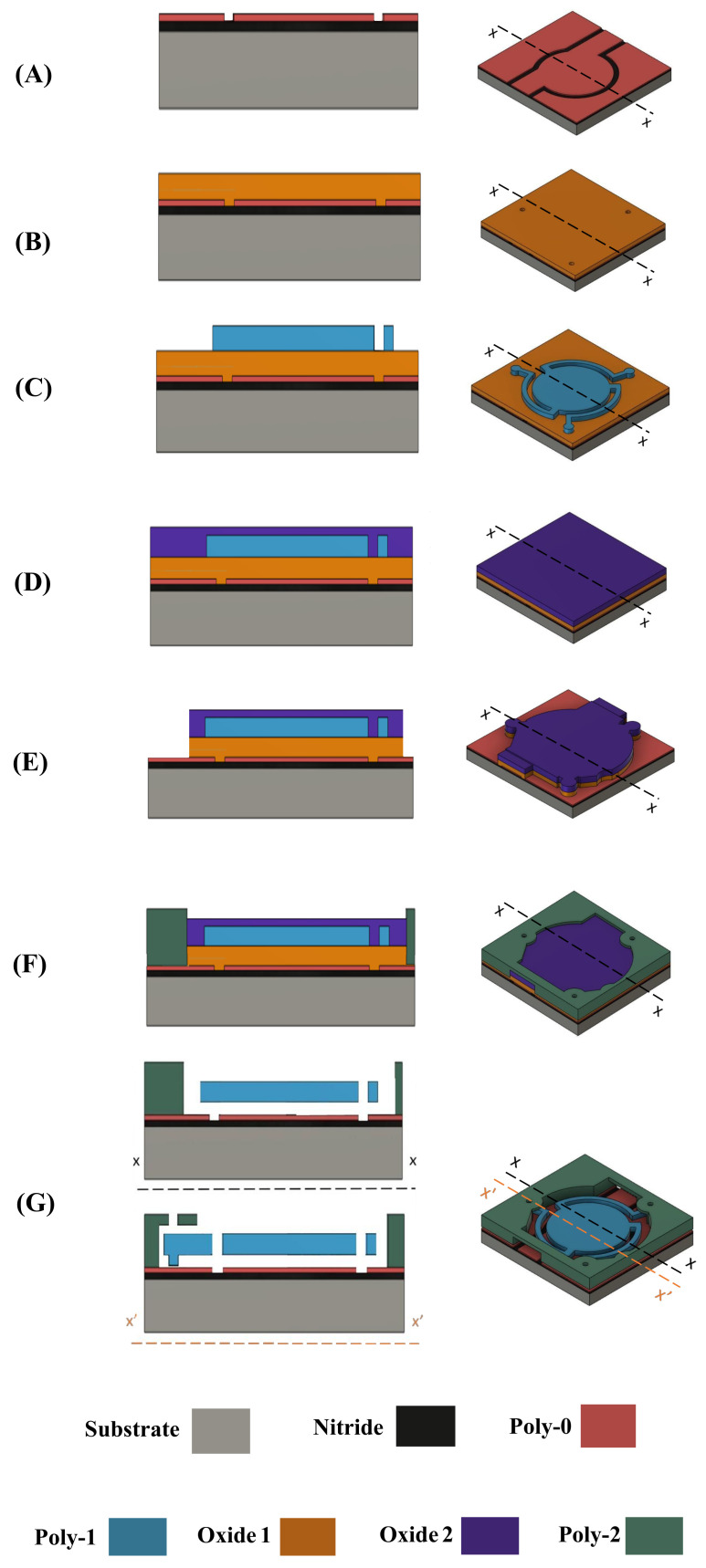
Cross-section *x*–*x* (**left**), and 3D (**right**) views of the different steps of the proposed fabrication process flow.

The CMUT and the array were designed according to PolyMUMPs guidelines and were taped out for implementation. Figure 3 shows cross-sections and 3D views of each main step of the fabrication process for a CMUT device.

The process begins with a silicon wafer substrate covered by a 600 nm silicon nitride (SiN) layer. A 500 nm layer of polysilicon (PolySi) is deposited and patterned with reactive ion etching (RIE) on the SiN layer and will serve as the lower electrode layer. Patterning divides the Poly-0 layer in three separate electrodes as visible in (A). The middle part serves as the fixed electrode, whereas the two surrounding electrodes serve to contact and electrically connect with the stems of the CMUT membrane. Next, a first 2 μm sacrificial silicon dioxide (SiO_2_) layer is deposited and DIMPLES are lithographically patterned to define the stems of the CMUT membrane, as shown in (B). Poly-1 is then deposited over Oxide-1 and forms a 2 μm thick structural layer for the CMUT membrane. Patterning using RIE creates the novel structure of the CMUT as illustrated in (C). A second oxide layer (Oxide-2) of 0.75 μm is deposited as shown in (D). Next, to anchor the enclosure to the substrate, Oxide-1 and Oxide-2 layers are etched through in (E). Then, the second PolySi is deposited and patterned to form the enclosures to contain the stem supports, as shown in (F). Since the stem supports are not affixed to the layer below, the CMUT is free to slide on the horizontal plane of the substrate. The enclosure is designed to contain the stem support and restrict large motion of the structure across the plane, potentially allowing only for small sliding of the stem base. Finally, the entire substrate is placed in hydrofluoric acid in order to dissolve the sacrificial oxide layers, releasing the finished product as shown in (G). PolyMUMPs’ layer thicknesses are given in Table 2.

**Figure 4:**
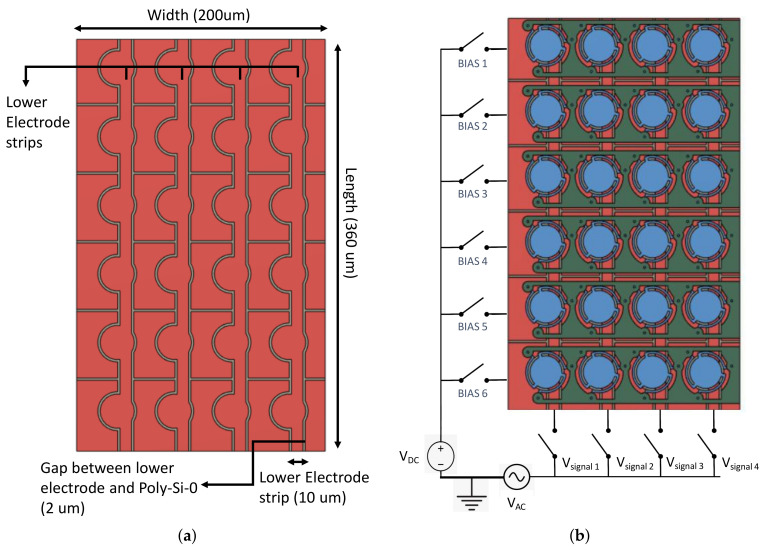
Top View of CMUT array. (**a**) Array dimension; (**b**) full array topology and electrical actuation setup.

**Figure 5:**
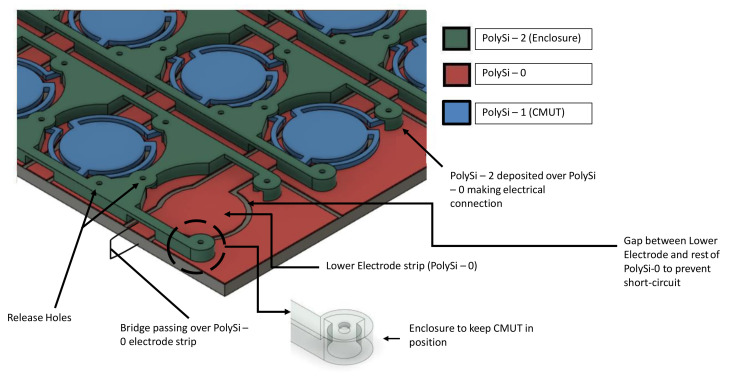
3D view of the CMUT array system with PolyMUMPS layers.

As a proof of concept, we propose the design of a CMUT array consisting of 6 × 4 CMUT units occupying only a 360 × 200 μm substrate footprint (Figure 4a). As illustrated in Figure 4b, Poly-0 serves as the main electrical routing layer for the array. Longitudinal conductive strips carry the AC signal to the bottom electrode of the selected column(s). The remainder of the layer is arranged into islands that are electrically connected in horizontal rows by means of Poly-2 bridges joining adjacent stem enclosures. These row electrodes serve to direct DC bias and AC ground to the bottom electrode of CMUT elements on the selected row(s). Electrical connection from the Poly-0 island to the Poly-1 membrane is ensured by mechanical contact with the support stems. The array routing strategy makes it possible to activate each CMUT element independently by activating the appropriate row and column coordinates. Figure 5 details the geometric structure of the proposed CMUT array system.

### 3.2. CMUT Cell Design

**Figure 6:**
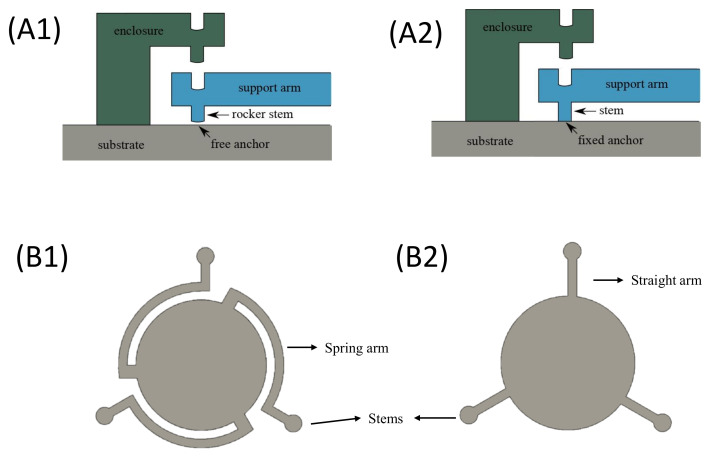
Illustration of the different anchoring (rocker (**A1**) and fixed (**A2**) stems) and support (spring (**B1**) and straight (**B2**) arms) configurations.

In the novel CMUT design, embodied in CMUT A of Figure 2, the suspended membrane is connected by spring arms (as shown in configuration B1 of Figure 6) to rocking support stems (as shown in configuration A1 of Figure 6). As highlighted in configuration A1, rocker stems are not fixed and are free to slide on the inferior layer, as highlighted in Figure 6. Conversely, a conventional CMUT (as embodied by CMUT D) is implemented by combining the fixed anchor stems of configuration A2 and the straight arms of configuration B2, as shown in Figure 6.

**Figure 7:**
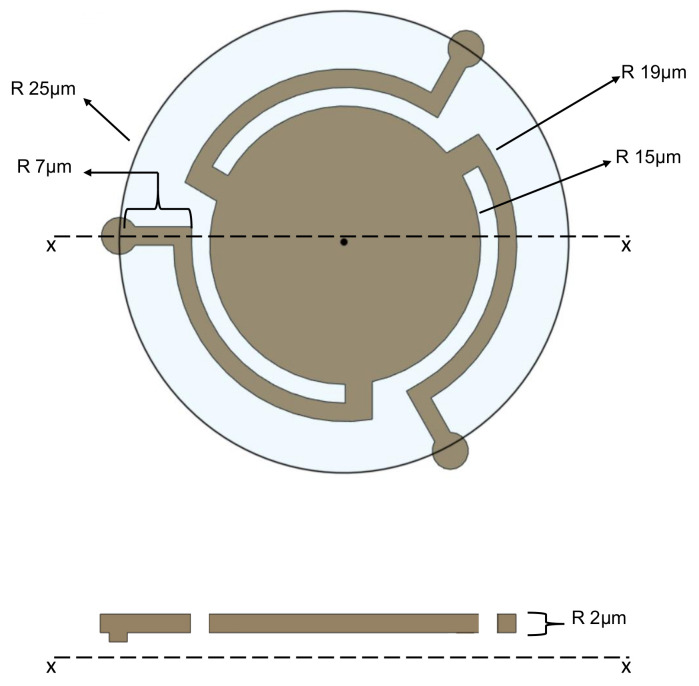
CMUT A and B cross-section.

During actuation of the device, due to the electrostatic force between the membrane and the fixed electrode caused by a DC bias, the membrane is attracted downwards toward the electrode. Downward deflection of the membrane also causes bending and twisting strain in the spring arms. Since the structure is supported at the stems, which are free to slide, but held down by static friction, only a small point or edge at the base of the stem will act as a pivot and will allow the device to rock about it. Static simulation was performed to evaluate the reaction forces on the rocker stem for CMUT A at 40 V DC electrostatic actuation. Normal (vertical) reaction force $F_{\mathrm{normal}}$ and radial outward force $F_{\mathrm{radial}}$ were determined to be 1.6 μN and 0.28 μN, respectively. The minimum static friction coefficient between silicon compounds can be estimated at 0.2 as per [20]. Since the resisting frictional force $\mu \times F_{\mathrm{normal}}$ is greater than $F_{\mathrm{radial}}$, it can be concluded that the rocker stem will remain static on the bottom surface, hence losing no energy to friction and avoiding any wear or tear. This novel approach is more efficient, since losses in the form of heat are greatly reduced [21,22], leaving more energy for the displacement of the membrane compared to devices with traditional anchoring (fixed stems). Since this approach minimizes effective stiffness, the resonance frequency is also lower compared to traditional anchoring for a similar footprint. The material properties and dimensions of the designed CMUT are provided in Table 3 and Figure 7.

## 4. Results and Discussion

In this study, the behaviour of CMUTs with different arm shapes and support types was investigated in terms of displacement and output pressure under DC and AC actuation. Simulations were performed in the air domain. The behaviour and performance of CMUTs were assessed using finite-element methods (FEM) models by means of the COMSOL Multiphysics software. Collapse voltage varied greatly between device types; therefore, the value of DC bias voltage was selected in accordance with the device having the lowest collapse voltage.

### 4.1. Static Simulation

**Figure 8:**
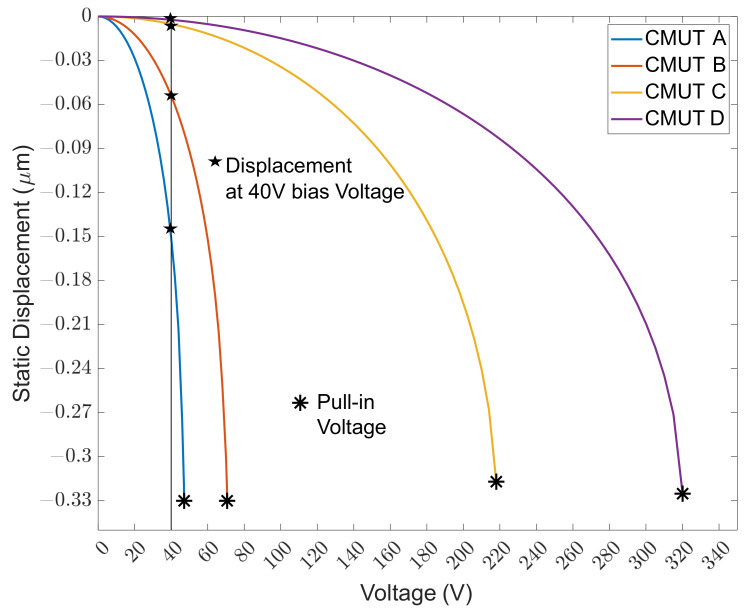
Static displacement vs. DC bias voltage.

**Figure 9:**
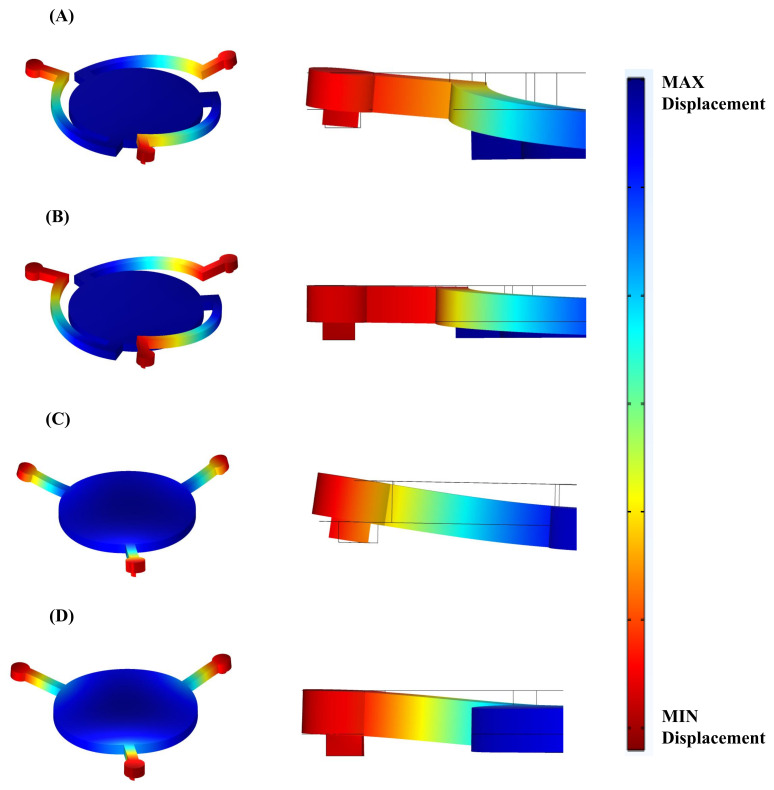
Scaled-up deformation of CMUTs under operation. (**A**) CMUT A. (**B**) CMUT B. (**C**) CMUT C. (**D**) CMUT D.

Collapse voltage was determined by plotting the displacement of a membrane with respect to DC biasing voltage for the devices, as shown in Figure 8, and determining the voltage above which the membrane displacement became unstable. The displacement value mentioned is for the center point of the membrane and corresponds to the maximum displacement of the membrane. Since the gap size for all the designed CMUTs is common at 1 μm, the cutoff voltages, marked by asterisks, occur for an identical displacement. Collapse voltages of 46 V, 71 V, 223 V, and 326 V were noted for CMUTs A, B, C and D, respectively. A significant reduction in collapse voltage can be appreciated between CMUTs C/D and CMUTs A/B in Figure 8 because of the introduction of spring arms which significantly reduce structural stiffness. This means that modifying the connecting arm structure has a greater impact on collapse voltage and resonance frequency than changing the support type, although both methods can be combined for greater effect. At 40 V, the static displacement value was 0.1508 μm, 0.0541 μm, 0.00507 μm, and 0.00231 μmm for CMUT A, B, C and D, respectively. Figure 9 shows CMUTs under operation at 40V DC bias and validates superior bias displacement for CMUT A and CMUT B under the same DC bias.

### 4.2. Configuration and Parameters for Dynamic Simulation

CMUTs have several damping loss mechanisms such as squeeze film damping, support losses and thermoelastic damping (TED). Air squeeze-film damping is produced when a DC voltage is combined with an AC signal across the electrodes of the CMUT, which leads to the membrane (piston) vibrating at the frequency of the AC excitation [23]. Squeeze-film damping is dominant in MEMS devices, and its influence becomes significant with decreasing device dimensions [24] as well as with lower resonance frequency devices [25,26]. A thin film-damping boundary condition was applied on the bottom side of the vibrating-membrane model, with the distance to the base selected as the electrode gap. Modeling of the squeeze-film damping effect is limited to the region between membrane and bottom electrode. Thermoelastic damping (TED) is the energy loss due to temperature gradients created by cyclic stress during the CMUT’s motion. Therefore, squeeze film damping is preeminent in CMUT A and CMUT B while TED is expected to be stronger in CMUT C and CMUT D. Thermal expansion multiphysics was added to the COMSOL model to take TED effects into consideration, but they were found to have a negligible impact. The Q factors established in simulations are, thus, dominated by the modeling of the squeeze-film damping effect.

In order to simulate a rocker support boundary condition for CMUT A and C, the inner edge of the bottom of the stem was constrained to a zero prescribed displacement in the direction perpendicular to the substrate. A fixed anchoring boundary condition for CMUT B and D was achieved by selecting the full bottom surface of the stem as a fixed constraint. A point at the center of the vibrating membrane was selected to plot in time the various displacement profiles. The bias voltage was applied to the device using a progressive ramp function at time t equal to zero in order to avoid potential instabilities that could be caused by a very fast step. After the transient displacement due to bias voltage settled down to its final position, the AC signal was applied with a smoothly increasing amplitude.

### 4.3. Harmonic Simulation

**Figure 10:**
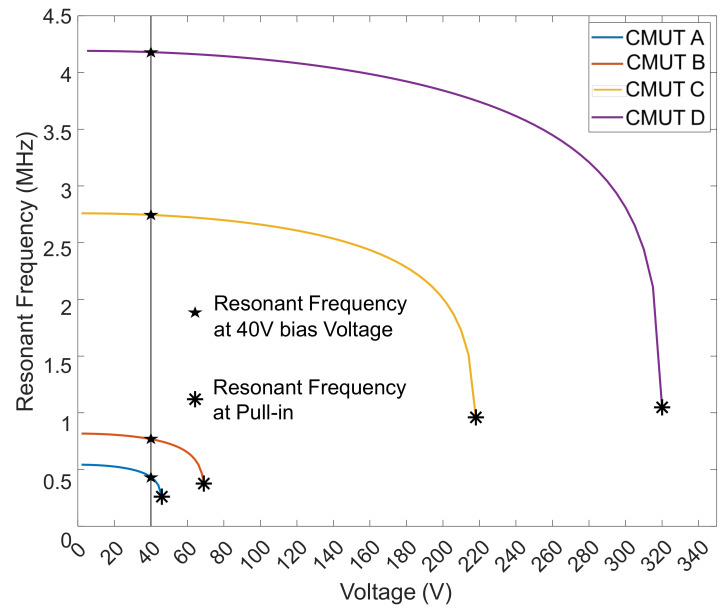
DC voltage vs. resonant frequency for CMUTs.

**Figure 11:**
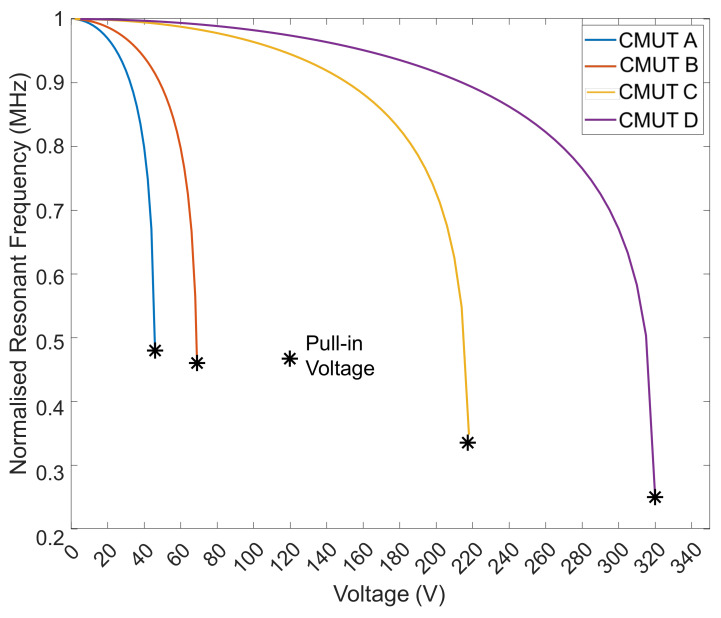
DC voltage vs. normalized resonant frequency for CMUTs.

The DC bias of each CMUT was increased until collapse to measure the decrease in the resonance frequency due to spring softening phenomena. A sharp drop in resonant frequency vs. DC bias can be seen near collapse voltage in Figure 10. Even though CMUT A has a resonance frequency about 2× lower than that of CMUT B and 6× less than that of CMUT C, it exhibits a similar relative frequency tunability range (between 50% and 60%) as the other two (Figure 11). CMUT D shows a slightly higher tunability of about 70%, although its resonance frequency is nearly ten times that of CMUT A. For a 40 V bias, resonance frequencies of 0.443 MHz, 0.787 MHz, 2.84 MHz, and 4.16 MHz were noted for CMUTs A, B, C and D, respectively. The deformation mode shapes of the four CMUT types are shown in Figure 10. Q factor for each of the four CMUTs was evaluated to be 18, 17, 16, and 15 for CMUTs A, B, C, and D, respectively. The Q factor is inversely proportional to the damping of the CMUT membrane: since there is increased damping from CMUT A to D, it is natural for the Q factor to decrease.

### 4.4. Dynamic Displacement

**Figure 12:**
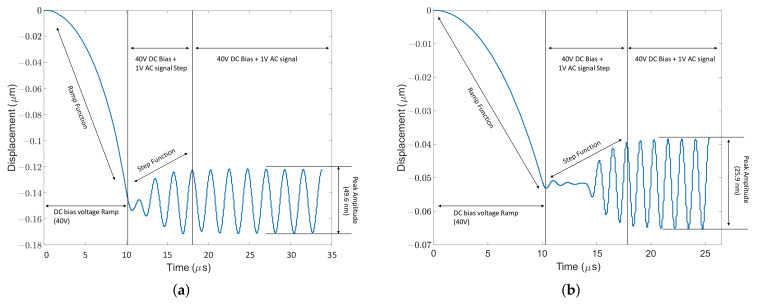

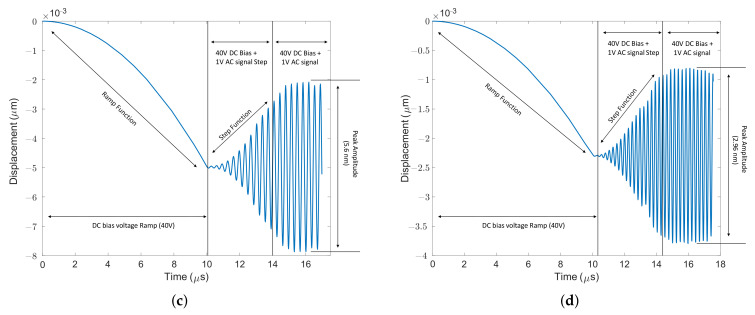
Peak-to-peak amplitude of CMUTs. (**a**) Peak-to-peak amplitude of CMUT A; (**b**) peak-to-peak amplitude of CMUT B; (**c**) peak-to-peak amplitude of CMUT C; (**d**) peak-to-peak amplitude of CMUT D.

**Figure 13:**
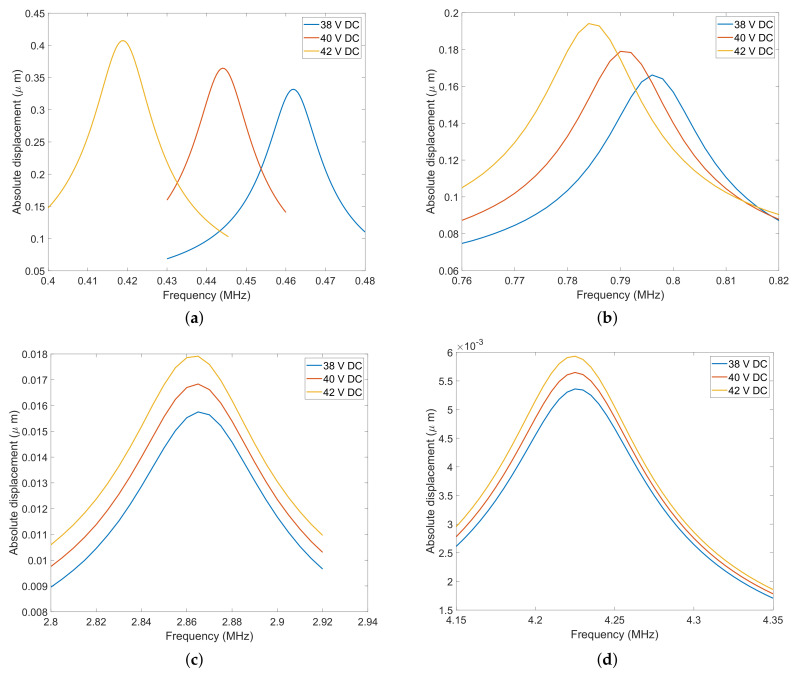
Frequency response of CMUTs.(**a**) Frequency response of CMUT A; (**b**) frequency response of CMUT B; (**c**) frequency response of CMUT C; (**d**) frequency response of CMUT D.

Displacement vs. time graphs are shown for CMUTs A, B, C, and D in Figure 12. The peak-to-peak displacement decreases from CMUT A to CMUT D because the common DC bias voltage selected (40 V) is closest to the collapse voltage of CMUT A and farthest from that of CMUT D. The peak-to-peak displacement for all CMUTs is provided in Table 4, proving that it is crucial to operate a CMUT close to its collapse voltage for maximum power efficiency. Since portable CMUT applications are restricted to low voltages, the novel designs of CMUTs A/B with their low collapse voltage can find utility. The frequency response of CMUTs was simulated as a function of different bias voltages. Figure 13 shows the frequency response of the four CMUT topologies at 38 V, 40 V and 42 V DC bias.

### 4.5. Dynamic Pressure

**Figure 14:**
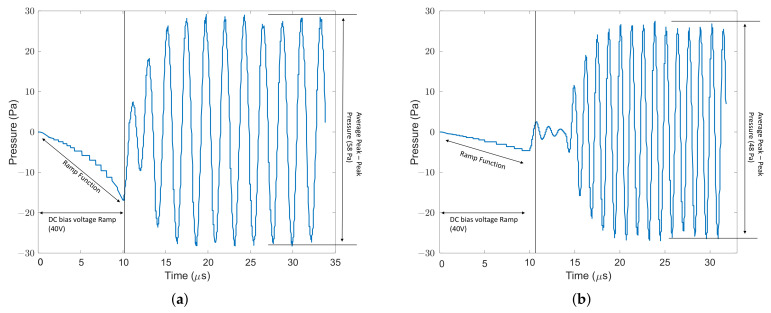

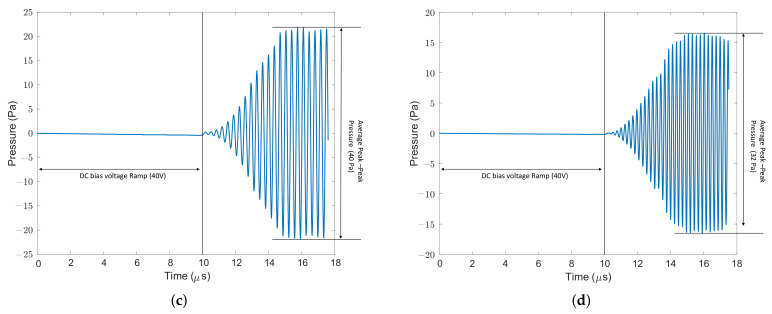
Pressure on CMUTs surfaces. (**a**) Pressure at CMUT-A surface; (**b**) Pressure at CMUT-B surface; (**c**) Pressure at CMUT-C surface; (**d**) Pressure at CMUT-D surface.

Far field pressure was measured 6 mm away from the CMUT surface using exterior field calculation in COMSOL. Pressure generated by an array of 50 × 50 CMUTs was also calculated by linearly adding pressure from each CMUT, assuming in-phase operation, with the results shown in Table 4. Acoustic pressure at the surface of a membrane shows similar behaviour as its displacement. Acoustic pressure is calculated by Equation (12), where *P* is pressure, *Z* is the acoustic impedance of the medium through which the sound wave travels, and *V* is the vibrating membrane velocity. The output pressure *P* is proportional to the displacement based on acoustic wave theory [17]. Instantaneous pressure vs. time graphs is shown for CMUTs A, B, C, and D in Figure 14. Peak pressure is determined for each of the four CMUTs by inputting maximum membrane velocity in Equation (12) and is also provided in Table 4.

(12) P=Z×V

### 4.6. Displacement Efficiency

Displacement efficiency is calculated as the peak–peak displacement per AC signal volt. The maximum displacement efficiency for the four designed CMUTs is 49.6 nm/V, 25.9 nm/V, 5.6 nm/V, and 2.96 nm/V for CMUT A, B, C and D, respectively. In comparison, CMUTs designed by [27,28] showed a displacement efficiency of 3.51 nm/V and 9 nm/V, respectively.

### 4.7. Electromechanical Coupling Coefficient

In general, transducers convert energy from one domain to another. For acoustic transducers, the conversion is between the electrical and mechanical domains. The electromechanical coupling coefficient $Kt^{2}$ is defined as the amount of mechanical energy delivered to the load to the total energy stored in the device:

(13) $$Kt^{2}=\frac{E_{Mech}}{E_{Total}}$$

where, *E_total_* = *E_elec_* + *E_mech_*. This coefficient is an important parameter in ultrasonic transducer design because it measures the degree of energy coupling (efficiency) between the electrical and mechanical domains. The coupling coefficient of a CMUT is derived by calculating free and fixed capacitance in [29] and is defined as

(14) $$Kt^{2}=\frac{2x(t)}{g-x(t)}$$

**Figure 15:**
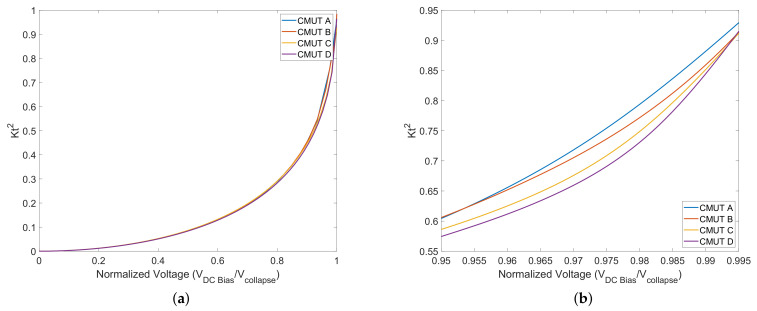
Electromechanical-coupling coefficient as function of voltage normalized to collapse voltage. (**a**) Electromechanical-coupling coefficient between 0–1 *V_normalized_*; (**b**) electromechanical -coupling coefficient between 0.95–1 *V_normalized_*.

The above result is important because near the collapse point (*g*/3), the coupling coefficient approaches unity, indicating that all the energy in one domain is converted to the other domain. Electromechanical coupling coefficient as function of voltage normalized to collapse voltage is shown in Figure 15. Figure 15a shows the electromechanical coupling coefficient as a function of bias voltage for the four CMUT types. The maximum difference between coefficients for CMUTS is close to the collapse voltage where they are operated. Higher electromechanical-coupling coefficient values can be seen for CMUT A followed by CMUT B, C, and D in Figure 15b. This is a result of CMUT A being able to convert electrical energy to mechanical (acoustic) energy more efficiently due to reduced energy losses in the anchors. The figure confirms that CMUT A is a more efficient device.

## 5. Conclusions

Four alternate CMUT transducers were designed and their realization using the PolyMUMPs technology was discussed. The proposed device improvements utilize a reduced stiffness design where curved beam supports are used instead of the typical straight version, and rocker stem supports instead of fixed anchors. These modifications allow for a greater displacement of the CMUT membrane for the same electrostatic force as well as increased sensitivity. In addition, they enable a reduction in the CMUT collapse voltage, and, by extension, the lowering of operating bias voltage to levels that are reasonable for portable applications. Moreover, the proposed design results in a significant reduction in the resonant frequency and increased frequency tunability for just a very small absolute variation in DC bias voltage. At the bias voltage of 40V, CMUT A had a static displacement about 2.5× of CMUT B, 30× of CMUT C and 65× of CMUT D, respectively. Resonant frequency at 40 V for CMUT A were noted as 2× lower than CMUT B, 6× lower than CMUT C and 9× lower than CMUT D. A similar trend is visible for pressure and peak–peak displacement. Electromechanical-coupling coefficient graphs show a steeper slope for CMUT A compared to the rest, confirming more displacement efficiency. CMUT A has several advantages over the conventional CMUT design (CMUT D) when operated at low bias voltage such as high tunability and use in portable applications. This work demonstrated the potential utility of the novel rocker stem support, which could eventually be applied to various MEMS device types. Physical devices have been taped out for fabrication and will be characterized experimentally to confirm this work’s conclusions.

## Figures and Tables

**Table 1:** Topology of designed CMUTs.

	Connecting Arm	Anchoring
CMUT A	Spring arm	Rocker support
CMUT B	Spring arm	Fixed anchoring
CMUT C	Straight arm	Rocker support
CMUT D (conventional)	Straight arm	Fixed anchoring

**Table 2:** PolyMUMPs Layers.

Layer	Thickness (um)
Nitride	0.6
Poly-0	0.5
Oxide-1	2
Poly-1	2
Oxide-2	0.75
Poly-2	1.5

**Table 3:** Material properties and dimensions of the novel CMUTs.

PolySi Young’s modulus (E)	158 GPa
PolySi Modulus of rigidity (G)	69 GPa
PolySi Poisson’s ratio (*v*)	0.22
PolySi density (*σ*)	2230 kg/m
Rocket stem height (h)	1 μm
Rocker stem radius (rd)	1 μm
Diaphragm radius (r)	30 μm
Diaphragm thickness (t)	2 μm
Gap between electrode (g)	1 μm

**Table 4:** CMUT output characteristics at 40 V DC bias.

CMUT	P-P Pressure (Pa)	P-P Disp (nm)	Biased Resonant Frequency (MHz)	Pressure at 6 mm (Pa)	Pressure at 6 mm for Array (Pa)
A	58	49.6	0.443	0.075	187.5
B	48	25.9	0.787	0.05	125
C	40	5.6	2.84	0.02	50
D	32	2.96	4.16	0.017	42.5

## Data Availability

Not applicable.
